# New horizons in improving research capacity in English care homes for older adults

**DOI:** 10.1093/ageing/afaf211

**Published:** 2025-08-03

**Authors:** Elisa Ruiz-Burga, Paul Flowers, Zoe Fry, Mike Slator, Lauren Hague, Martin Green, Adam Lee Gordon, Ann-Marie Towers, Martin Knapp, Claire Surr, Laura Shallcross

**Affiliations:** Institute of Health Informatics, University College London, London, United Kingdom of Great Britain and Northern Ireland; Department of Psychological Sciences and Health, University of Strathclyde—School of Psychological Sciences and Health, Glasgow, Scotland, United Kingdom of Great Britain and Northern Ireland; The Outstanding Society, England, United Kingdom of Great Britain and Northern Ireland; The Outstanding Society, England, United Kingdom of Great Britain and Northern Ireland; Care England, London, England, United Kingdom of Great Britain and Northern Ireland; Care England, London, England, United Kingdom of Great Britain and Northern Ireland; Queen Mary University of London—Wolfson Institute of Population Health, London, United Kingdom of Great Britain and Northern Ireland; Barts Health NHS Trust—Academic Centre for Healthy Ageing, London, England, United Kingdom of Great Britain and Northern Ireland; King‘s College London—Health and Social Care Workforce Research Unit, The Policy Institute, London, United Kingdom of Great Britain and Northern Ireland; Care Policy Evaluation Centre, London School of Economics, London, England, United Kingdom of Great Britain and Northern Ireland; NIHR UCLH—Biomedical Research Centre, London, United Kingdom of Great Britain and Northern Ireland; School of Health, Leeds Beckett University—Centre for Dementia Research, Leeds, United Kingdom of Great Britain and Northern Ireland; NIHR UCLH—Biomedical Research Centre, London, United Kingdom of Great Britain and Northern Ireland

**Keywords:** ageing, research capacity, care homes, residents, older adult

## Abstract

As the care home sector expands over time, the health and social care needs of both residents and staff intensify and diversify. These sector-wide changes call for significant growth in research capacity to deliver useful, pertinent and timely evidence.

In this paper we highlight growing pressures in the care home sector, and the major and enduring barriers to conducting research across that sector, within homes and amongst staff, drawing primarily on our experiences conducting public health research in England. These obstacles include a lack of national infrastructure, tradition and culture, and underdeveloped systems to reimburse providers for the staff and other costs associated with research delivery.

Finally, we detail short, medium and long-term actions that could enable the growth of research capacity across the sector. These include leveraging political will, remunerating and crediting research champions, and establishing a feedback loop to showcase the contribution of research in improving both quality of care and resident outcomes. Our suggested actions focus on what would be required to build research capacity in care homes in England; although these also have relevance in other countries where there is a need and wish to build research capacity in adult long-term care facilities.

## Key Points

- Research capacity in care homes has been significantly hampered by a combination of longstanding and newly arising challenges in the social care (long-term care) sector.
- COVID-19 exposed care home residents’ vulnerability, demanding innovative interventions without compromising quality of care and damaging quality of life.
- Building research capacity requires a multi-level and phased strategy for lasting transformation.
- A logic model is a useful approach to outline the necessary steps for transforming research in care homes into sustainable and scalable research delivery.

## Introduction

The COVID-19 pandemic highlighted the vulnerability of care home residents to adverse outcomes following infection. Across many countries, this prompted calls for innovative, proportionate public health interventions to safeguard residents and staff from future infections, outbreaks and pandemics without damaging quality of care or compromising quality of life [1]. Addressing this evidence gap requires high-quality interventional research studies at scale that consider residents’ physical and mental health and wellbeing, while aligned with the needs, priorities and business models of the sector. However, there continue to be multiple, complex barriers to research delivery in these settings.

**Figure 1:**
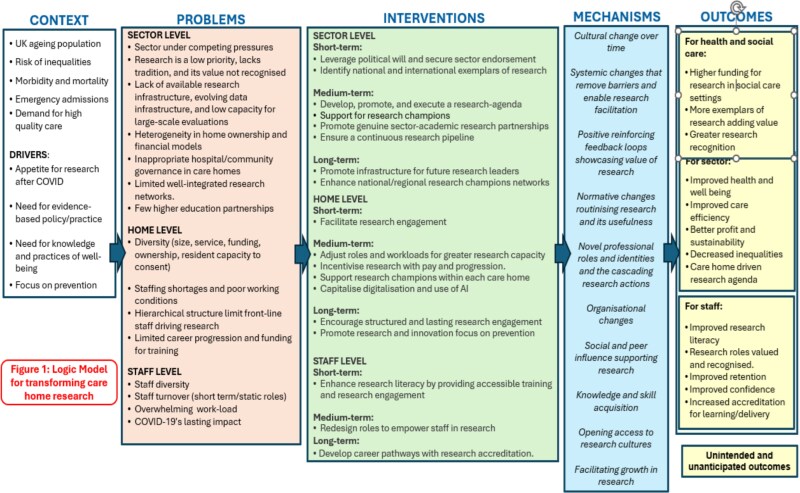
Logic model for transforming care home research.

Before the pandemic, large-scale research studies on infection or other diseases in social care (long-term care) settings were rare, and typical public health systems to monitor disease in the general population usually did not extend to care homes. In England, our research group set up the Vivaldi studies during the COVID-19 pandemic to address this evidence gap. Between May 2020 and August 2023, we used a range of study designs (survey, cohort study, data linkage, clinical trial) to generate evidence on health outcomes in residents, including disease burden, reinfection risk, effectiveness of vaccination and strategies to limit transmission of infection in care homes for older adults [2, 3]. Importantly, our research was delivered in partnership with care providers and policy communities, enabling us to deliver research at pace and scale in a manner not previously seen in the care sector. In this article, we reflect on the published literature and our learning from Vivaldi study. We propose a logic model setting out what would be required to transform health and public health research and sustain research delivery in care homes for older adults in the short, medium and long term (see Figure 1, the logic model). This model was developed following an ‘away day’ attended by members of the research team, including people with experience of working in the sector and relatives of care home residents. The logic model and manuscript were refined with input from academic collaborators listed as authors of the paper bringing expertise in the care of older adults and adult social care. This same model could support social care research, but this would be contingent on social care outcomes being collected and recorded in digital social care records.

## Context: the need to increase research capacity

The UK’s rapidly ageing population, combined with increasingly complex care needs, is driving demand for health and social care. The number of people aged over 85 is projected to double by 2041 and triple by 2066, with total costs of social care predicted to escalate from £18.3 billion in 2018 to £35.5 billion in 2038 [4]. The UK care home sector is diverse, with a range of providers and funding models, including self-funding, charitable support, public funding [from local government and the National Health Service (NHS)] [5]. For example, people entering care homes tend are increasingly likely to be frail with complex needs due to a deliberate policy to support people in their own homes for longer, reduced access to home-based social care and higher financial thresholds for publicly funded care [6–9]. There are also marked inequalities in access to care, quality of care, and outcomes, with disparities often linked to socioeconomic status, age, disability and location [10].

The lack of research capacity in social care severely undermined efforts to generate evidence-based policy to inform the public health response to the COVID-19 pandemic [11]. Despite a funding landscape that committed to a growing social care research capacity in recent years (including in care homes), COVID-19’s legacy has made it even more challenging for many care providers to participate in research by increasing their financial vulnerability, making it yet more difficult to recruit and retain staff. Pressures on the NHS have also increased, making it harder for residents to access healthcare. Staff who survived the COVID pandemic and continue to work in the sector have experienced psychological trauma [12]. This is often associated with a sense of unease about authority (e.g. Government, academia, the NHS) due to policy decisions imposed on staff in the care sector during the pandemic (e.g. mandatory vaccinations) [13, 14], and anxiety triggered by rapidly changing policy guidance.

Interventional research studies in care homes and the wider social care sector are comparatively underdeveloped, frequently yielding inconclusive results, with barriers to research implementation that threaten study validity [15]. The evidence that the sector requires must be relevant at scale, address challenges of importance to the sector, while prioritising the needs and preferences of residents, families and staff. It should address sector-wide inequalities, residents’ increasingly complex physical and mental health needs, challenges to their wellbeing, and residents’ interactions with staff and their physical and social environments. It should also drive improvements in healthcare quality by exploring innovative care models and improvements to surveillance programmes. The changes proposed in the logic model, and specifically access to routinely collected data to measure healthcare outcomes, create potential for a range of studies to prevent avoidable hospital admissions for residents—from falls prevention to medication errors and vaccination [16]. Delivering this kind of research requires transformations in research capacity to address the multiple barriers to conducting studies of widespread relevance across the sector.

## Problem: what prevents or undermines delivery of research in care homes?

Multiple, long-standing and emerging challenges hinder the development of research capacity within care homes in England and internationally [17, 18].

*Sector-level barriers:* Complex regulatory frameworks, funding constraints and under-staffing limit the sector’s capacity for research [10, 19]. Additionally, the care sector’s primary focus on operational demands, combined with lack of established research culture, and the sense that research is often ‘done to’ rather than ‘done with’ the sector reduces the likelihood that research questions address care home priorities. Thus, care home owners, providers and staff do not always recognise the benefits of engaging with research [20]. Historically, the Care Quality Commission (CQC), which regulates care delivery in England, and the Department of Health and Social Care, the central government department responsible for social care, have not supported the required step change in research capacity. Consequently, there is little incentive for care providers to establish research governance frameworks or prioritise research training for staff [21]. By contrast, research is part of the NHS constitution [22]. NHS Trusts receive support and financial incentives to recruit patients to research from the National Institute for Health and Care Research (NIHR). These structures are comparatively underdeveloped in community and care home settings. A further barrier is lack of research leadership roles within the sector, and few (albeit notable) examples of capacity-building via academic and care partnerships [23]. The NIHR Enabling Research in Care Homes (ENRICH) network provides a toolkit to help care homes make the most of research and helps researchers to deliver care home studies. However, ENRICH staff are not embedded in care homes, limiting their ability to support study set-up and delivery [24]. Research governance and assurance frameworks, developed for healthcare settings, can be challenging to apply in care homes, creating additional barriers to research delivery, particularly for studies that span community and hospital settings. Research frameworks vary across countries, making it difficult to scale research models or studies internationally. There is also lack of clarity about definitions and approval processes required for quality improvement projects versus research studies, particularly when studies span both domains, creating unnecessary delays when negotiating ethical and governance approvals.

*Home-level barriers*: Care home heterogeneity includes aspects such as size, resident population and care needs, funding models, ownership and workforce [25], posing challenges when attempting to control for variation between homes within a trial or implementing standardised interventions [26]. It can also undermine efforts to recruit a representative sample of facilities, impacting on subsequent generalisability and relevance of findings. The challenges of staff shortages, poor working conditions, low pay and high staff turnover (30% annually) are well-documented [10, 19]. Some providers and managers remain reluctant to engage in research, seeing few short- or long-term benefits, despite compelling evidence underscoring the need for further research to address uncertainties and needs in care homes [27]. This is an important barrier because care home managers and staff play critical gatekeeping roles in supporting researchers to access potential participants [28]. Staff members’ willingness to engage with research is likely to influence their awareness of research opportunities, and their willingness to support residents to participate in studies [29, 30]. Additionally, care homes that are financially unstable are unlikely to prioritise research participation. Even if the benefits of research are understood, they may be outweighed by set-up costs and the steep learning curve associated with complex ethical and governance approvals [29]. With the exception of research conducted during the COVID-19 pandemic, few organisations within the sector have first-hand experience of how research can drive improved care and outcomes for residents, staff and businesses. Legitimate and salient concerns around the potential negative impacts of research [31], such as disruption to daily routines, increased stress for residents and staff, or reputational damage, are particularly pertinent if care providers cannot get indemnity for research studies.

*Staff-level barriers*: In addition to staff shortages and high staff turnover, the demanding nature of care home work also limits research capacity [32]. In contrast to NHS staff, research is not part of care workers’ roles, so line managers may be reluctant to release them to support research delivery [30, 33]. Care-specific qualifications are not a requirement for entry into the care home workforce and so many staff may not have the opportunity to access learning and development beyond induction and mandatory training. Consequently, very few care workers have research training or experience [34, 35]. On the other hand, workforce diversity and experience create opportunities to develop novel, scalable, pragmatic interventions building on staff members’ substantial knowledge, skills and acumen in care delivery. Research training and capacity-building for care home staff could provide new career pathways for them, helping to address sector-wide challenges in recruitment and retention. Care home staff-led research innovations could also support improved care delivery and efficiency.

## Interventions: what changes are needed to transform research capacity within the care home sector?

The complexity and interconnected nature of health and social care necessitate a multifaceted, staggered approach to build research capacity within the sector [36], recognising that changes at any single level are unlikely to yield the sustained cultural changes that are required [15, 37, 38].

### Sector-level changes

We are already starting to see ‘short-term’ strategic changes in approach to and support for research in the sector across a range of stakeholders. There have been increased calls and funding for social care and health research delivered in social care settings, including capacity-building initiatives [23, 39–41], alongside greater political will and recognition of the value of research by the CQC. Looking ahead, it will be essential to elevate the importance of care home research on the political agendas of all four UK nations. Further, aligning the priorities of policy organisations, media, Third Sector advocacy and umbrella bodies and the public is essential to support research capacity-building in care homes. To catalyse a cultural shift towards research, it is imperative to showcase exemplars of research that have directly benefited residents, staff, and organisations. We also need to demonstrate how research can support the economic sustainability of the sector, by quantifying the potential financial and reputational benefits for providers of research participation (including direct income from research, cost savings from more efficient care and/or reduced staff sickness, improved CQC ratings enhancing marketability to future residents and public sector purchasers). Studies undertaken in the COVID-19 pandemic highlighted how research can inform policy; we must capitalise on this momentum and continue to emphasise the clear link between research, policy changes, improved care, health and well-being, and cost-savings for providers, local authority purchasers and the NHS [42, 43]. In this manner, models of success that matter to residents, families, care providers and carers must be defined and widely celebrated.

In the ‘medium-term’*,* we require new research infrastructure that empowers research champions to generate and execute a care sector-driven research agenda. This requires securing funding [44], responding to gaps in knowledge, identified needs within the care sector, and priorities of funding bodies. Likewise, there is a need to foster and sustain engagement from residents, relatives and care provider organisations, and to develop a robust research pipeline with dedicated funding for academic-care sector collaborations [29]. Highlighting successful partnerships between higher education institutions and the sector can inspire further models of collaboration.

‘Long-term strategies’ include identifying, retaining and developing future research leaders and key opinion leaders within the sector to champion research and advocate for culture shift. Enhancing national/regional networks to support research champions and knowledge exchange is crucial. A more robust national research infrastructure, building on ENRICH but embedding capacity within care homes, is also essential. This requires dedicated funding, training programmes, and collaborative networks [45, 46].

**Home-level changes**: A key ‘short-term’ strategy for care homes is to involve residents, their families, care home staff, academics, clinicians, and other relevant stakeholders in building and shaping research agendas [47–49]. For example, co-designing a research agenda and planning sensible approaches to develop research processes, including ethical considerations, is important to ensure responsible care home research. ‘Medium-term actions’ include allocating time for research within staff roles and providing physical space and resources to support research activities. Work is required to consider the different roles of care home staff, how they might be involved in research, the differing training needs of each group, opportunities for peer-to-peer learning, and how to recognise, reward and align these new skills with care workers’ career progression. Finally, there are major opportunities and challenges to capitalise on rapid digitisation of social care (70% of providers use a digital tool to plan and record care) [50, 51], e.g. using natural language processing to harness data recorded in free text within these systems to support research [52]. Likewise, there are many ways in which technological innovations can enhance quality of care, for example by using virtual reality to improve awareness of the risk of falls [53]. However, guidance and processes are needed to support care homes to select technologies that benefit their residents and are cost-effective, further highlighting the need for research studies that address effectiveness (from the perspective of health and social care) and implementation. In ‘the long-term’*,* we need to provide systematic opportunities for staff, residents and relatives to play leading roles in shaping future research priorities. By enabling care sector-led research and innovation, we can also support Government policy to shift from disease treatment to disease prevention, benefiting residents, families and care home staff.

### Staff-level changes

‘Short-term’ strategies will be mostly focused on increasing research awareness and literacy using a diverse and flexible training programme. Complementary strategies include incentivising staff participation in research through remuneration and accrediting opportunities for research learning, engagement and delivery to support role development, career progression and improved pay [54, 55]. The diverse cultural and educational backgrounds of staff members and their language skills are a major asset to enable interventions to be adjusted to specific settings. Also, as the sector’s research capacity matures, there will inevitably be a need to reinforce research culture, disseminate good practice and ensure peer-to-peer learning. The ‘medium term’ will involve specific strategies to empower and train staff according to their roles. For instance, at managerial level, leaders should champion research and innovation, allocate resources and forge partnerships to drive implementation of evidence-based practices. This aspect may help increase staff motivation to participate in research training to facilitate professional development and to empower them to foster a culture of research. ‘Long-term strategies’ will include promoting new career pathways to incentivise research engagement and care sector-led research studies in partnership with academia.

## Unintended and adverse consequences

We acknowledge that this represents a bold vision for developing research capacity in the sector. The use of routine data from residents who lack capacity to consent raises complex ethical and governance challenges which need to be set against the potential benefits to this population of increased access to research. There is also a risk that the additional workload associated with research participation inadvertently increases financial strain on the sector by adding to staff workload. Perhaps most importantly, failure to engage effectively with the sector throughout this initiative could generate more disenchantment with research, making it even more challenging to deliver future studies in social care settings. On the other hand, our proposed approach could foster unforeseen positive outcomes, such as a stronger sense of community and collaboration amongst stakeholders working in different sectors (social care, NHS, local authorities, universities and policy communities) through their collective participation in research, potentially leading to better integration of health and social care and catalysing new career development opportunities for staff.

## Conclusion

We have set out an ambitious vision for how to build and sustain research capacity in care homes for older adults, adopting a multi-faceted, staged approach (see Figure 1) that draws on previous research and our experiences of delivering studies in care homes, particularly during the COVID-19 pandemic. Although our work to date has focused on older adults, we anticipate that it would be feasible to adopt a similar approach to enable research delivery in settings serving younger adults.

Our first step towards realising our vision has been to establish the ‘Vivaldi Social Care (VSC) project’ [56]*,* which builds on learning from the original Vivaldi study conducted in more than 300 homes. Vivaldi benefitted from strong support from the care sector due to its focus on Covid-19 and momentum created by the pandemic. It seems unlikely that an equivalent sized study could have been established in a similar timescale to address other less urgent health and/or social care priorities but learning from Vivaldi is pertinent for building research capacity in care homes today. In the UK, the most notable and recent attempt to establish a large care home study was PROTECT-CH [57], a platform trial, which aimed to evaluate prophylactic therapies for Covid-19 in residents but stopped early due to the development of the Covid-19 vaccine. Internationally, there are several examples of interventional and observational studies enabled by access to large, established networks of care providers and/or routinely collected data. Taken together, these studies demonstrate increased appetite for large-scale studies in care homes and the potential of care home networks and routine data to enable research delivery [58, 59].

The VSC project comprises a network of research-motivated care homes underpinned by linked data, and has been coproduced by academics, policy officials, people who live and work in care homes and organisations that represent the care sector [60, 61]. Our aim is to reduce the impact of infections, outbreaks and antimicrobial resistance in care homes for older adults by designing research in a way that removes barriers to participation for providers, residents and staff. Around 700 care homes are taking part, having agreed to share limited data on their residents. Over the next 5–10 years, our goal is to use this infrastructure to enable the sustained and efficient delivery of clinical and other trials and other designs of research studies in care homes, by working with the care sector, residents and families, the national and international research community, charities, research funders and Government to enact the multi-levelled changes outlined in our logic model.

We have outlined a range of interventions needed to realise the vision outlined in this article, but perhaps the biggest challenge for the research community is how to sustain a pipeline of funded research studies and thus demonstrate the feasibility, affordability and cost-effectiveness of consolidating research activity within a care home network. This will require a critical mass of inter-disciplinary researchers (social scientists, clinicians, public health specialists, behavioural scientists, economists, statisticians, trialists) working in social care settings. It is encouraging to see recent progress in these areas, including ring-fenced NIHR funding calls for social care research and funded training opportunities and internships for social care staff [62]. However, more could be done to attract researchers from other disciplines to work in social care by highlighting the opportunities to deliver inter-disciplinary studies that have major, beneficial impacts on residents, staff, families and the wider health and care system. To sustain the research pipeline, it is also critical to maximise the impact and visibility of studies in social care, by selecting research outcomes that resonate with diverse stakeholders (especially residents, staff, relatives, providers, the NHS, and public sector purchasers of care). This means giving equal weight to health and social care outcomes and, wherever possible, quantifying the economic benefits of research participation for care providers, local authorities and the NHS.
